# Effects of 4-hydroxy-2,5-dimethyl-3(2H)-furanone supplementation on growth performance, serum antioxidant capacity, rumen fermentation characteristics, rumen bacterial quorum sensing, and microbial community in Hu sheep

**DOI:** 10.5713/ab.24.0683

**Published:** 2025-02-27

**Authors:** Chuanpei Fu, Jing Ge, Mingren Qu, Kehui Ouyang, Qinghua Qiu

**Affiliations:** 1 Jiangxi Province Key Laboratory of Animal Nutrition and Feed, College of Animal Science and Technology, Jiangxi Agricultural University, Nanchang, China

**Keywords:** 4-Hydroxy-2,5-Dimethyl-3(2H)-furanone, Antioxidant Capacity, Quorum Sensing, Rumen Fermentation Characteristic, Rumen Microbial Community

## Abstract

**Objective:**

This study aimed to investigate the effects of 4-hydroxy-2,5-dimethyl-3(2H)-furanone (HDMF) on growth performance, serum antioxidant capacity, rumen fermentation characteristics, rumen microbial LuxS/AI-2 quorum sensing, and community composition in Hu sheep.

**Methods:**

Twenty-two male Hu sheep with similar body weights (19.97±0.41 kg) and ages (66.75±2.11 days) were evenly divided into two groups: the control group, receiving a basal diet (n = 11, CON), and the treatment group, receiving the basal diet supplemented with an additional 1.0 g/d of HDMF (n = 11, HDMF).

**Results:**

The results showed no significant differences in average daily gain, average daily dry matter intake, and feed conversion ratio between the HDMF and CON groups (p>0.05), but numerical improvements of 11.12%, 4.55%, and 7.56% were observed, respectively. Compared to the CON group, the HDMF group exhibited elevated levels of serum glutathione peroxidase and superoxide dismutase, and decreased levels of malondialdehyde and reactive oxygen species, as well as a reduced oxidative stress index (p<0.05). Dietary HDMF supplementation did not affect rumen fermentation characteristics, except for the proportion of valerate (p<0.05). HDMF supplementation increased the AI-2 signaling molecules concentration and biofilm formation in the rumen fluid (p<0.05). No substantial differences were seen in the richness and evenness of rumen microbes between the HDMF and CON groups. Principal coordinates analysis and nonmetric multidimensional scaling analyses revealed an obvious overlap between the two groups, and analysis of similarities also indicated no significant differences (R = 0.0116, p = 0.303). Subsequent species annotation and biomarker analysis revealed that the HDMF group reduced the abundances of *Shuttleworthia*, *Eubacterium eligens* group, and *Erysipelotrichaceae UCG 002*, and increased the abundances of *Riknellaceae RC9 gut* group, *Prevotellaceae UCG 003*, *Desulfobulbus*, *Methanobrevibacter*, and *Selenomonas ruminantium*.

**Conclusion:**

This study indicates that HDMF supplementation enhances the body’s antioxidant capacity and increases the abundances of certain disease-resistant bacteria by promoting AI-2 signaling molecules and biofilm formation, thereby ultimately contributing to the enhanced growth performance of Hu sheep.

## INTRODUCTION

Feed additives are pivotal in boosting the productivity and health of ruminants. They enhance rumen fermentation, optimize nutrient absorption, and bolster overall animal health, which in turn leads to improved growth, reproductive success, and superior quality of products [1]. Integral to the digestive process, rumen microorganisms adeptly convert complex plant fibers into absorbable nutrients [2]. They are critical in transforming feed into meat, milk, and other products, thereby significantly influencing the efficiency and sustainability of ruminant agriculture [3]. Strategically manipulating these microbes via targeted feed additives presents a promising path to increase nutrient utilization and production yields, while also potentially mitigating environmental impacts [4]. Therefore, the development of feed additives designed to regulate rumen microbiota is a viable and promising approach to boost ruminant productivity and promote sustainability.

Quorum sensing (QS) is a communication mechanism used by bacteria to assess their population density and synchronize their behavior based on the presence of autoinducers [5]. This sophisticated system is crucial for the adaptation and survival of bacterial communities within their environments [5]. The LuxS/autoinducer-2 (AI-2) system is a predominant form of QS in rumen microbial communities, with the enzyme LuxS producing AI-2, a signaling molecule that facilitates interspecies communication [6,7]. The presence of AI-2 synthase and receptor genes in many rumen microbial genomes indicates that LuxS/AI-2 QS plays a significant role in the rumen ecosystem, potentially influencing feed degradation and nutrient conversion efficiency. Liu et al [8] found that AI-2 mediated QS can induce biofilm formation, and under the action of these biofilms, rumen microorganisms can effectively degrade fibrous materials in feed. Huws et al [9] also observed that a distinct biofilm forms in the rumen within the first 5 minutes of digesting roughage. During the primary colonization stage, the expression of the *luxS* gene increased among rumen microbes, while it decreased during the secondary colonization stage. Metagenomic studies have revealed that dairy cows with low feed efficiency had a richer QS metabolic pathway in the rumen [10]. Furthermore, Atlanderova et al [11] reported that QS inhibitors increased the levels of volatile fatty acids (VFA), ammonia nitrogen, and total nitrogen within the rumen fluid of beef cattle, while decreasing the levels of non-protein nitrogen, thus enhancing nitrogen utilization efficiency. These studies suggest that QS modulators may serve as potential feed additives for improving ruminant production performance and enhancing animal health.

4-Hydroxy-2,5-dimethyl-3(2H)-furanone (HDMF) is recognized as a common modulator in the LuxS/AI-2 QS system among bacteria [12]. It is a naturally occurring flavor compound found in various fruits and heat-processed foods, such as beef broth, roasted almonds, and coffee [13]. Martinelli et al [14] found that synthetic furanones could mimic the activity of N-acylhomoserine lactones, thereby inducing the formation of violacein. However, HDMF has also been reported to act as an inhibitor of QS in certain bacteria, such as *Pseudomonas aeruginosa* and *Trueperella pyogenes*, suggesting its potential as a therapeutic agent for combating bacterial infections [15,16]. Moreover, HDMF also has the ability to suppress the expression of virulence factors in *Pseudomonas aeruginosa*, including biofilm formation, motility, and the QS system [15]. This indicates that HDMF could diminish the virulence of certain bacteria, potentially resulting in a reduced prevalence of diseases in ruminant animals. The findings from these reports demonstrate that the precise impact of HDMF may vary depending on the specific bacterial species involved. Considering the diverse array of species within the rumen, the overall influence of HDMF on the performance and health of ruminants remains to be fully elucidated.

Therefore, a 75-day animal feeding trial was conducted to investigate the effects of HDMF on various aspects of ruminant health and productivity. The trial aimed to assess the impact of HDMF on growth performance, serum antioxidant capacity, rumen fermentation characteristics, rumen microbial diversity, QS and community composition. The goal was to evaluate the feasibility and underlying mechanisms of HDMF as a feed additive to enhance ruminant production. The study hypothesized that HDMF may promote the growth of sheep by enhancing serum antioxidant capacity and modifying rumen microbial communities and QS. As the first research to explore the application of HDMF in animal production, it aims to pave the way for new insights into the use of QS modulators to advance animal production.

## MATERIALS AND METHODS

### Animal care

In compliance with the highest standards of animal care and welfare, the study was conducted under the strict supervision and authorization of the Committee for the Care and Use of Experimental Animals at Jiangxi Agricultural University, as per protocol number JXAULL-2021036.

### Animals, diets, and experimental design

A total of twenty-two healthy male Hu sheep, with similar body weight (19.97±0.41 kg) and age (66.75±2.11 days), were divided evenly into two groups. One group was fed a basal diet (n = 11, CON), while the other group received an additional 1.0 g per day of HDMF on top of the basal diet (n = 11, HDMF). The ratio of concentrate to forage in the basal diet was 70:30, with the specific ingredient composition and chemical components as shown in Table 1. HDMF was purchased from Hubei Yongkuo Technology Co., Ltd. (Tianmen, China), with a purity of 99.5%. The entire trial lasted for 75 days, including a 15-day adaptation period and a 60-day experimental period. The sheep were housed individually, with free access to feed and a clean supply of drinking water throughout the trial. Given the requirement to monitor growth performance in this trial, and taking into account that each group comprised 11 biological replicates, we opted not to employ a crossover design.

### Sample collection

During the trial, the dry matter intake was recorded daily. The body weights were measured on two consecutive days at the beginning and end of the trial. Two days before the trail’s end, blood was collected from the jugular vein into blood collection tubes and centrifuged at 2,012×g for 15 minutes to obtain serum samples. Before morning feeding, rumen contents were collected using the oral intubation method as described by Paz et al [17], and the the first 50 mL was discarded to reduce saliva contamination. The collected rumen contents included both liquid and solid phases. After passing through four layers of gauze and being divided into centrifuge tubes, they were immediately frozen in liquid nitrogen and then transferred to a laboratory freezer at −80°C for storage.

### Parameter determination

The rumen pH value is measured immediately after the rumen fluid is obtained using a portable pH meter (Testo 206, Testo AG, Schwarzwald, Germany). The ammonia nitrogen (NH_3_-N) concentration was determined following the protocol established by Broderick and Kang [18], utilizing the phenol-hypochlorite colorimetric method. The concentration of microbial crude protein (MCP) was measured using the Folin phenol method, which is an adaptation of Lowry’s assay, as delineated in the work of Makkar et al [19]. In this study, the VFA assessed included acetate, propionate, isobutyrate, butyrate, isovalerate, and valerate, with the collective sum of isobutyrate, isovalerate, and valerate categorized as branched-chain VFA (BCVFA). Each VFA component was identified by its relative retention time and quantified against a standard curve, both established under identical operational conditions. The analysis was conducted on a gas chromatograph (GC-2014, Shimadzu Corporation, Kyoto, Japan), following the scheduled procedures and parameter settings outlined in Qiu et al [20].

Serum parameters included cortisol and antioxidant indicators, which are used to assess stress status and antioxidant capacity, respectively. In this study, the antioxidant indicators measured included total antioxidant capacity (T-AOC), glutathione peroxidase (GSH-Px), superoxide dismutase (SOD), malondialdehyde (MDA), and reactive oxygen species (ROS). All assays were conducted strictly according to the instructions provided with the corresponding assay kits from the Beijing Sinouk Institute of Biological Technology (Beijing, China). The oxidative stress index (OSI) was calculated by dividing ROS by T-AOC, serving as a comprehensive measure of antioxidant capability.

### DNA extraction, sequencing and data analysis

Genomic DNA from rumen fluid samples was extracted utilizing a Bacterial DNA Kit (OMEGA, Omega Bio-Tek, Norcross, GA, USA), employing a two-step protocol that included bead-beating for initial lysis [17]. The integrity and concentration of the DNA were assessed using 1% agarose gel electrophoresis and a spectrophotometer (NanoDrop 2000, Thermo Fisher Scientific, Wilmington, DE, USA), respectively. A total of 22 samples, each exhibiting high purity and quality, were forwarded to Allwegene Gene Technology Co., LTD (Nanjing, China) for further amplification and sequencing. The V3-V4 hypervariable regions of the 16S rRNA gene were targeted for amplification to characterize bacterial diversity and community structure, utilizing barcoded primers as reported by Wei et al [21]: 338F (5′-ACTCCTACGGGAGGCAGCAG-3′) and 806R (5′-GGACTACNNGGGTATCTAAT-3′). The amplification protocol and reaction conditions were in accordance with those detailed by Qiu et al [20]. Polymerase chain reaction amplicons were inspected on a 1% agarose gel, purified using Agencourt AMPure XP Kits (Beckman Coulter, Brea, CA, USA), and subsequently used to construct libraries for microbial diversity sequencing. High-throughput sequencing was conducted on an Illumina NextSeq 2000 PE300 platform, generating paired-end reads. The raw sequencing data have been archived in the Sequence Read Archive (SRA) under accession number PRJNA1158230.

The sequencing data were subjected to analysis using Quantitative Insights Into Microbial Ecology (QIIME 2) [22]. The paired-end reads were merged using Fast Length Adjustment of Short reads (FLASH, version 1.2.11) [23], with a maximum mismatch rate of 0.10 and a minimum overlap of 10 base pairs. Sequences were filtered out if they did not meet the following criteria: length between 200 and 500 bp, quality score of at least 20, absence of ambiguous bases or chimeric sequences, and match to primer sequences and barcode tags. The sequences that passed the quality control were denoised into amplicon sequence variants (ASVs) utilizing the Deblur algorithm within QIIME 2. ASVs from all samples were rarefied to a common depth of 31,113 reads, yielding an average of 265 ASVs per sample. Taxonomic classification of each ASV was conducted using the Ribosomal Database Project (RDP) Classifier at a 70% confidence threshold, aligning with the bacterial SILVA 138 database.

### Statistical analysis

The normality of the data distribution was assessed using the Shapiro-Wilk test. As all data, except for the microbiome data, met the assumptions of normality, an independent samples t-test was performed using SPSS software (version 20; IBM Corporation, Armonk, NY, USA) to compare the mean differences between the CON and HDMF groups. Statistical significance was set at p<0.05, with a trend was noted p-values between 0.05 to 0.10 (0.05≤p<0.10). For the microbiome data, Wilcoxon rank-sum test was used to compare the differences between the CON and HDMF groups. QIIME 2 was employed to calculate five alpha diversity indices—Chao 1, observed species, phylogenetic diversity (PD) whole tree, Shannon index, and Simpson index—to characterize the richness and evenness of the rumen bacterial community. To elucidate the differences and similarities between the CON and HDMF groups, principal coordinates analysis (PCoA) and nonmetric multidimensional scaling (NMDS) were performed using R software (version 4.0.2), based on Bray-Curtis distances. An analysis of similarities (ANOSIM) was also conducted to explore the community structure similarities between the CON and HDMF groups, utilizing the vegan package within R software. Furthermore, an Linear Discriminant Analysis (LDA) Effect Size (LEfSe) analysis was introduced to identify biomarkers within each group. This analysis was carried out using LEfSe software, with an LDA score threshold set at 3.0 [24]. Phylogenetic Investigation of Communities by Reconstruction of Unobserved States (PICRUSt) was utilized to predict the metagenomic contributions of the detected communities, thereby elucidating the intrinsic ruminal functions of the bacterial microbiota.

## RESULTS

### Growth performance

Table 2 presents the effect of dietary HDMF supplementation on growth performance of Hu sheep. Compared to the CON group, dietary supplementation with HDMF did not result in significant differences in final body weight (FBW), average daily weight gain (ADG), average daily dry matter intake (DMI), or feed to gain ratio (F/G) (p>0.05). However, there was a numerical increase of 4.72% in FBW, 11.12% in ADG, and 4.55% in DMI. Additionally, there was a 7.56% decrease in the F/G.

### Serum antioxidant capacity

The effect of dietary HDMF supplementation on serum antioxidant capacity of Hu sheep is listed in Table 3. Dietary supplementation with HDMF significantly increased the levels of GSH-Px and SOD, reduced the levels of MDA and ROS, and ultimately lowered the OSI (p<0.05). Additionally, there was a trend suggesting that HDMF supplementation might decrease cortisol levels (p = 0.055).

### Rumen fermentation characteristics

The effect of dietary HDMF supplementation on rumen fermentation characteristics of Hu sheep is shown in Table 4. Dietary HDMF supplementation had no significant impact on ruminal pH value, concentrations of MCP, NH_3_-N, total VFA or individual VFA (p>0.05). In contrast, valerate concentration tended to decrease in the HDMF group (p = 0.096), while the proportion of valerate was significantly lower in the HDMF group compared to the CON group (p<0.05).

### Quorum sensing molecules and biofilm formation

**Figure 1:**
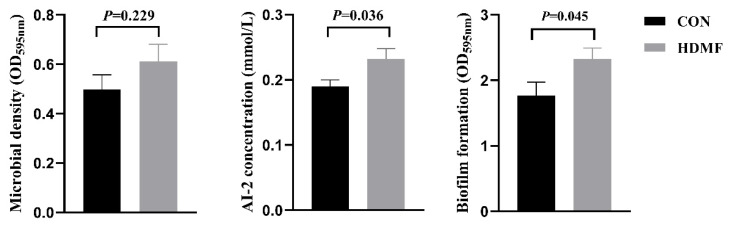
Effect of dietary HDMF supplementation on the concentration of rumen quorum sensing signaling molecule and biofilm formation in Hu sheep. HDMF, 4-hydroxy-2,5-dimethyl-3(2H)-furanone.

As shown in Figure 1, the AI-2 concentration and the level of biofilm formation were higher in the HDMF group compared to the CON group (p<0.05). No significant differences were observed in microbial density (p>0.05).

### Rumen bacterial diversity and community composition

HDMF supplementation did not alter rumen bacterial richness or evenness, as indicated by the Chao1, observed species, PD whole tree, Shannon index, and Simpson index (p>0.05, Table 5). A total of eleven phyla were observed with relative abundances greater than 0.1%. Bacteroidota, Firmicutes, and Proteobacteria occupied the top three positions, accounting for 40.18%, 37.86%, and 19.23%, respectively. However, no significant differences were observed between the CON and HDMF groups for any of these phyla (p>0.05, Table 6). Twenty-four genera were observed with relative abundances greater than 0.5%, with *Prevotella* and *Succinivibrio* occupying the top two positions, accounting for 32.75% and 13.45%, respectively. The relative abundance of *Eubacterium eligens group* was significantly lower in the HDMF group compared to the CON group (p<0.05, Table 7).

### Beta-diversity of rumen bacteria

**Figure 2:**
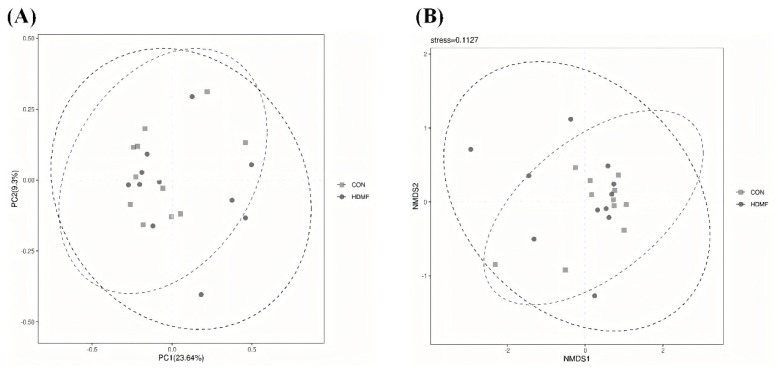
Effect of dietary HDMF supplementation on beta-diversity of the rumen bacteria in Hu sheep. (A) Principal coordinates analysis (PCoA); (B) non-metric multidimensional scaling (NMDS). HDMF, 4-hydroxy-2,5-dimethyl-3(2H)-furanone.

As shown in Figure 2, both PCoA (Figure 2A) and NMDS (Figure 2B) plots clearly reveal overlaps between the HDMF and CON groups. Additionally, ANOSIM found no significant differences in the microbial composition between the HDMF and CON groups (R = 0.0116, p = 0.303).

### Biomarker analysis

**Figure 3:**
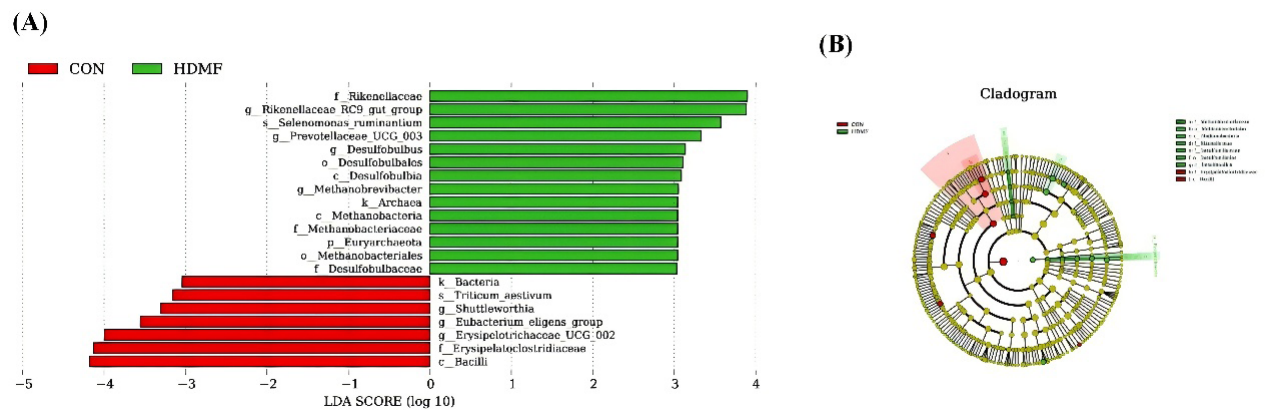
Effect of dietary HDMF supplementation on the discriminative bacterial communities at different taxonomic levels. (A) Linear discriminant analysis (LDA); (B) Cladogram. HDMF, 4-hydroxy-2,5-dimethyl-3(2H)-furanone.

The LDA distribution bar graph (Figure 3A) indicates that a total of twenty-one species were observed with an LDA score greater than 3.0 and a significance level of p<0.05. In the CON group, the biomarkers with significant discriminative power were Bacilli, Erysipeloclostridiaceae, *Shuttleworthia*, *Eubacterium eligens group*, and *Erysipelotrichaceae UCG 00*2. In the HDMF group, they were Euryarchaeota, Desulfobulbia, Methanobacteria, Desulfobulbales, Methanobacteriales, Desulfobulbaceae, Rikenellaceae, Methanobacteriaceae, *Riknellaceae RC9 gut group*, *Prevotellaceae UCG 003*, *Desulfobulbus*, *Methanobrevibacter*, and *Selenomonas ruminantium*. Cladogram (Figure 3B) derived from the LEfSe analysis revealed the similar biomarkers with LDA distribution bar graph.

### Predicted functions of ruminal bacterial microbiota

This study identified 29 gene families in the rumen fluid samples, 23 of which had relative abundances greater than 0.1% (Table 8). Gene families related to the metabolism of cofactors and vitamins, carbohydrate metabolism, and amino acid metabolism were predominant in the rumen microbiome of both HDMF and CON groups. However, no significant differences were observed among these three gene families or any other gene families examined in this study (p>0.05).

## DISCUSSION

### Effect of dietary supplement of HDMF on growth performance and serum antioxidant capacity of Hu sheep

HDMF exhibits antimicrobial, anti-inflammatory, and antioxidant properties in the biopharmaceutical field [25,26], making it a promising candidate for animal production. HDMF has a sweet caramel-like odor and is one of the major compounds generated by the Maillard reaction [27]. However, no increase in DMI was observed in this study, suggesting that this caramel-like flavor may not a taste preferred by the Hu sheep. While the numerical enhancements in ADG and feed conversion ratio were observed with dietary HDMF supplementation, they did not reach statistical significance. The brevity of the feeding period may have obscured these differences. Echoing this, Li et al [28] reported that there was only a mere numerical increase in growth performance within the initial 60 days of glutamate supplementation, with growth significantly accelerating after 90 days of feeding Hu sheep. A longer feeding period is required to confirm the growth-promoting effects of dietary HDMF supplementation, as the current short-term feeding has already shown numerical improvements.

Serum hormone levels and antioxidant capacity provided a good explanation for the numerical improvements in growth performance. Cortisol is a crucial glucocorticoid in animals, playing a pivotal role in stress response, metabolism, immune modulation, and cognitive function [29]. It helps animals adapt to various challenges by regulating energy availability and maintaining homeostasis [30]. However, high cortisol levels signify a state of physiological arousal, preparing the animal to cope with perceived threats [30]. Dietary supplementation with HDMF tended to decrease the level of cortisol, implying that HDMF can alleviate chronic stress in animals. GSH-Px and SOD are integral components of the antioxidant defense system in animals [31,32]. GSH-Px uses glutathione to reduce hydrogen peroxide and lipid peroxides, thereby preventing oxidative damage to cellular components [31]. On the other hand, SOD catalyzes the conversion of superoxide radicals into molecular oxygen and hydrogen peroxide, which GSH-Px then further metabolizes [31]. The coordinated action of these enzymes is essential for maintaining cellular redox balance and mitigating the harmful effects of ROS. Higher levels of GSH-Px and SOD were observed in the HDMF group, likely due to the inherent antioxidant properties of HDMF itself. MDA, a byproduct of lipid peroxidation, serves as a biomarker for oxidative stress in animals [33]. The lower levels of MDA in the HDMF group indicate that the addition of HDMF alleviated stress, which corresponds to the trend of decreased cortisol levels. ROS, naturally produced in cells, play a dual role in physiological processes and pathogenesis [34]. Essential for cell signaling and homeostasis, ROS are also implicated in various diseases when present in excessive levels, leading to oxidative damage to lipids, proteins, and DNA [34]. The HDMF group exhibited lower levels of ROS, indicating that dietary supplementation with HDMF mitigated oxidative damage in the body. This reduction in overall oxidative stress is further supported by a decreased OSI. The data suggest that HDMF supplementation in the diet can enhance the antioxidant capacity and reduce oxidative stress in Hu sheep, potentially improving their growth performance.

### Effect of dietary supplement of HDMF on rumen fermentation characteristics

Rumen fermentation is a critical biological process in ruminants, pivotal for the efficient degradation of fibrous plant material [35]. The complex microbial activity within the rumen transforms cellulose into VFA, which are essential for providing the primary energy substrates for the ruminants [36]. Furthermore, these rumen microorganisms are crucial in nitrogen metabolism, as they convert dietary protein into NH_3_-N and amino acids. These nitrogenous compounds are then utilized for the synthesis of MCP, which is vital for the protein nutrition of ruminants [37]. In this study, dietary supplementation with HDMF did not alter most of the rumen fermentation characteristics, except for the concentration and proportion of valerate. Valerate is principally produced through the microbial fermentation of dietary carbohydrates and branched-chain amino acids [36]. However, propionibacteria also convert lactic acid into propionic and valeric acids via the propionic acid metabolic pathway [38]. Both the concentration and proportion of valerate decreased after dietary supplementation with HDMF, possibly due to the antimicrobial effect of HDMF leading to a reduction in the activity of propionibacteria. This reduction can also be indirectly corroborated by the decrease in the numerical values of propionate concentration and proportion. These results suggest that the dietary addition of HDMF may inhibit the activity of some VFA-producing microorganisms, thereby reducing the production of valerate. However, the specific mechanisms remain to be elucidated through further investigation.

### Effect of dietary supplement of HDMF on rumen bacterial quorum sensing and community structure

In the rumen, the LuxS/AI-2 QS system, identified as the dominant QS mechanism through metagenomic and metatranscriptomic studies, is crucial for interspecies communication among rumen bacteria [6,8]. AI-2, the central and distinctive signaling molecule of the LuxS/AI-2 QS system, allows bacteria to sense its concentration and synchronize their collective actions, such as adjusting cell density and initiating biofilm formation [9,39]. The study revealed that dietary addition of HDMF led to a marked enhancement in AI-2 levels and biofilm formation. These findings suggest that HDMF stimulates rather than suppresses QS among rumen microbial communities, contradicting earlier literature [12]. The discrepancy in outcomes might stem from the fact that previous studies identifying HDMF as an inhibitor of LuxS/AI-2 QS focused on specific bacterial strains *in vitro*, whereas the rumen houses a diverse microbial community, which could respond differently to HDMF. Furthermore, the complex structure of biofilms not only bolsters bacteria’s resilience against external environmental challenges but also facilitates the adhesion of rumen microorganisms to feed particles [39]. This promotes the efficient breakdown of feed, thereby enhancing feed conversion efficiency [9,39]. The notable increase in biofilm formation observed in the HDMF group sheds light on the numerical upgrades in feed conversion rates and the concurrent increase in serum antioxidant activity following the dietary addition of HDMF.

Alpha diversity metrics quantify the richness and evenness of species within a specific sample or a single ecological community [40]. Alpha diversity plays a pivotal role in preserving the stability and functionality of the rumen ecosystem, significantly impacting the efficiency of feed digestion and nutrient absorption in ruminant animals [41]. There were no significant differences in either species richness or evenness between the CON and HDMF groups, suggesting that dietary supplementation with HDMF did not impact the alpha diversity of the rumen microbial community. Taxonomic annotation offers a more holistic perspective on microbial community structure across various levels. At the phylum level, no significant differences were detected between the CON and HDMF groups. At the genus level, the relative abundance of the *Eubacterium eligens group* in the HDMF group was significantly lower than that in the CON group. The *Eubacterium eligens group* is recognized for its pivotal role in producing short-chain fatty acids and its ability to utilize pectin, a complex carbohydrate found in plant cell walls [42]. This pectin-degrading ability is especially crucial in the rumen of ruminant animals, facilitating the fermentation process and bolstering the host’s energy yield. The relative abundance of the *Eubacterium eligens group* corresponded with the VFA production within the rumen fermentation characteristics, implying that the supplementation with HDMF might not be favorable for the efficiency of nutrient utilization. Nevertheless, this inference necessitates substantiation through further digestive experimentation.

The biomarker analysis revealed a higher discriminatory power at various taxonomic levels, spanning from phylum to species. Bacilli participate in the breakdown of hemicellulose and the utilization of polysaccharides [43], a function analogous to that of the *Eubacterium eligens group* previously discussed. This similarity further suggests that the dietary inclusion of HDMF could potentially exert adverse effects on nutrient utilization. The presence of Erysipeloclostridiaceae, *Shuttleworthia*, and *Erysipelotrichaceae UCG 002* has been correlated with inflammation or pathological conditions within the body [44,45]. Following the supplementation of HDMF, a notable reduction in their relative abundances was observed, which is indicative of an amelioration in the animal’s health. This observation aligns with the reduced cortisol levels and enhanced antioxidant capabilities reported earlier, pointing towards a positive shift in the physiological balance. The class of Desulfobulbia, along with its affiliated Desulfobulbales, Desulfobulbaceae, and *Desulfobulbus*, are sulphate-reducing bacteria involved in biofilm formation [46]. Within the HDMF group, the elevated relative abundance of these bacteria coincides with an increased level of biofilm formation, substantiating the notion that HDMF supplementation can bolster the animal’s resilience against external stressors. Members of the Methanobacteria, Methanobacteriales, Methanobacteriaceae, and *Methanobrevibacter*, which are part of the Euryarchaeota, contribute to greenhouse gas emissions by producing methane through the metabolism of organic compounds in anaerobic environments [47]. The activity of *Selenomonas ruminantium* plays a vital role in the rumen’s hydrogen gas recycling process, as they contribute to sustaining the low partial pressure of hydrogen, a condition that is essential for the functioning of methanogenic archaea [48]. Methane production signifies an extent of energy loss, as this energy could have otherwise been harnessed for growth and productivity [49]. The elevated relative abundance of these bacteria in the HDMF group implies a higher degree of energy loss, which is reflected in the comparatively reduced levels of VFA production within this group. The *Rikenellaceae RC9 gut group* stands out as a significant component of the Rikenellaceae family. It exhibits a positive correlation with the uptake and metabolic processing of VFA, thereby indirectly influencing the immune capabilities of the host [50]. Research also indicates a link between the *Rikenellaceae RC9 gut group* and the generation of metabolic byproducts by rumen microorganisms [51]. Predominantly, these byproducts are concentrated in metabolic pathways that include galactose metabolism, the biosynthesis of unsaturated fatty acids, and the breakdown of fatty acids, all of which are intricately connected to the host’s energy metabolism [51]. The studies suggest that the *Rikenellaceae RC9 gut group* is capable of bolstering the host’s resistance to diseases and the efficacy of VFA absorption. This could partially account for the observed improvement in the growth performance of sheep with the dietary addition of HDMF, despite no observed increase in VFA output. A wealth of research indicates that an increase in the ruminant’s antioxidant defenses and immune function is correlated with a higher relative abundance of *Prevotellaceae UCG-003* [52–54]. In addition, studies have revealed a significant positive correlation between *Prevotellaceae UCG-003* and feed efficiency [54,55]. The elevated relative abundance of *Prevotellaceae UCG-003* in the HDMF group provides a microbial rationale for the observed enhancement in immune function and improved feed efficiency resulting from dietary HDMF supplementation.

## CONCLUSION

In conclusion, dietary HDMF supplementation increased the serum levels of GSH-Px and SOD, while simultaneously reducing levels of MDA, ROS, and OSI. This supplementation has a subtle impact on rumen fermentation characteristics, particularly by reducing the proportion of valerate. Additionally, HDMF supplementation enhanced the levels of AI-2 signaling molecules and biofilm formation in the rumen fluid. HDMF supplementation did not affect the alpha diversity of the ruminal microbiota but influenced certain bacterial genera. Specifically, it reduced the relative abundances of *Shuttleworthia*, *Eubacterium eligens group*, and *Erysipelotrichaceae UCG 002*, and increased the relative abundances of *Riknellaceae RC9 gut group*, *Prevotellaceae UCG 003*, *Desulfobulbus*, *Methanobrevibacter*, and *Selenomonas ruminantium*. This study suggests that HDMF, acting as a LuxS/AI-2 QS regulator, bolsters antioxidant defenses, nutrient assimilation efficiency, and overall growth performance through the augmentation of AI-2 and biofilm formation. As the first study to investigate the effects of HDMF on ruminant growth performance from the perspective of rumen microbial QS, it provides innovative insights and new directions for research aimed at the optimization of production strategies in ruminant animals.

## Figures and Tables

**Table 1:** Ingredients composition and nutritional components of basal diet

Ingredient	Proportion (%)	Nutritional components	Value
Corn	45.04	Metabolizable energy (MJ/kg)	11.18
Soybean meal	18.72	Crude protein (g/kg)	150.81
Wheat bran	3.49	Neutral detergent fiber (g/kg)	313.93
Wheat straw	30.00	Acid detergent fiber (g/kg)	178.69
Calcium hydrogen phosphate	0.75		
Limestone	0.50		
Salt	0.50		
Premix^1)^	1.00		

1) Premix provided the following per kg of DM: 1400 mg of Fe, 1200 mg of Zn, 250 mg of Cu, 900 mg of Mn, 100,000 IU of vitamin A, 27,000 IU of vitamin D3, and 800 IU of vitamin E.

**Table 2:** Effect of dietary HDMF supplementation on growth performance of Hu sheep

Item	CON^1)^	HDMF^2)^	SEM	p-value
Initial body weight (kg)	19.99	19.94	0.582	0.952
Final body weight (kg)	35.57	37.25	0.934	0.227
Average daily gain (g/d)	259.57	288.43	15.323	0.244
Average daily dry matter intake (g/d)	1,041.00	1,088.39	33.757	0.297
Feed to gain ratio^3)^	4.10	3.79	0.243	0.416

1) CON indicates the control group, which was fed a basal diet.

2) HDMF represents the treatment group, which was received an additional 1.0 g per day of 4-hydroxy-2,5-dimethyl-3(2H)-furanone (HDMF) on top of the basal diet.

3) Feed to gain ratio was calculated by dividing the average daily dry matter intake by the average daily gain.

SEM, standard error of the mean.

**Table 3:** Effect of dietary HDMF supplementation on serum antioxidant capacity of Hu sheep

Item	CON^1)^	HDMF^2)^	SEM	p-value
Cortisol (ng/mL)	42.14	38.37	1.315	0.055
Total antioxidant capacity (U/mL)	35.35	37.53	1.266	0.278
Glutathione peroxidase (U/mL)	328.31^b^	349.20^a^	6.358	0.031
Superoxide dismutase (U/mL)	82.43^b^	88.08^a^	1.758	0.040
Malondialdehyde (nmol/mL)	3.51^a^	3.15^b^	0.094	0.023
Reactive oxygen species (EU/mL)	147.73^a^	123.79^b^	7.656	0.039
Oxidative stress index	4.18^a^	3.23^b^	0.246	0.014

1) CON indicates the control group, which was fed a basal diet.

2) HDMF represents the treatment group, which was received an additional 1.0 g per day of 4-hydroxy-2,5-dimethyl-3(2H)-furanone (HDMF) on top of the basal diet.

a,b In the same row, values with different superscripts differ significantly (p<0.05).

SEM, standard error of the mean.

**Table 4:** Effect of dietary HDMF supplementation on rumen fermentation characteristics of Hu sheep

Item	CON^1)^	HDMF^2)^	SEM	p-value
pH value	6.67	6.73	0.080	0.611
Microbial crude protein (mg/L)	1,415.91	1,650.22	135.69	0.255
Ammonia nitrogen (mg/dL)	4.85	4.47	0.74	0.716
Total volatile fatty acids (mmol/L)	36.91	33.78	3.079	0.480
Concentration (mmol/L)				
Acetate	21.80	20.57	1.886	0.649
Propionate	10.27	8.51	1.059	0.255
Isobutyrate	0.30	0.30	0.016	0.843
Butyrate	3.48	3.49	0.441	0.982
Isovalerate	0.42	0.40	0.050	0.739
Valerate	0.63	0.51	0.050	0.096
Branched-chain volatile fatty acids	1.36	1.20	0.091	0.243
Proportion (%)				
Acetate	59.38	60.88	1.282	0.421
Propionate	27.65	25.04	1.665	0.289
Acetate/propionate	2.23	2.63	0.199	0.189
Isobutyrate	0.88	0.94	0.070	0.570
Butyrate	9.18	10.45	0.751	0.264
Isovalerate	1.15	1.20	0.103	0.745
Valerate	1.76^a^	1.50^b^	0.086	0.041
Branched-chain volatile fatty acids	3.79	3.63	0.155	0.468

1) CON indicates the control group, which was fed a basal diet.

2) HDMF represents the treatment group, which was received an additional 1.0 g per day of 4-hydroxy-2,5-dimethyl-3(2H)-furanone (HDMF) on top of the basal diet.

a,b In the same row, values with different superscripts differ significantly (p<0.05).

SEM, standard error of the mean.

**Table 5:** Effect of dietary HDMF supplementation on rumen bacterial alpha-diversity metrics of Hu sheep

Item	CON^1)^	HDMF^2)^	SEM	p-value
Chao1	269.96	272.16	17.32	0.930
Observed species	269.00	271.45	17.19	0.921
PD whole tree	32.44	28.96	2.09	0.255
Shannon index	5.94	5.99	0.16	0.834
Simpson index	0.955	0.965	0.006	0.332

1) CON indicates the control group, which was fed a basal diet.

2) HDMF represents the treatment group, which was received an additional 1.0 g per day of 4-hydroxy-2,5-dimethyl-3(2H)-furanone (HDMF) on top of the basal diet.

SEM, standard error of the mean; PD, phylogenetic diversity.

**Table 6:** Effect of dietary HDMF supplementation on the rumen bacterial composition (phylum, >0.10%) of Hu sheep

Phylum name	CON^1)^	HDMF^2)^	SEM	p-value
Bacteroidota	37.87	42.48	3.695	0.389
Firmicutes	37.32	38.40	3.421	0.827
Proteobacteria	22.34	16.11	4.350	0.340
Actinobacteriota	0.82	0.82	0.243	0.995
Spirochaetota	0.21	0.72	0.202	0.150
Cyanobacteria	0.56	0.26	0.205	0.361
Fibrobacterota	0.20	0.28	0.129	0.663
Euryarchaeota	0.19	0.25	0.134	0.815
Patescibacteria	0.17	0.23	0.078	0.620
Desulfobacterota	0.21	0.17	0.047	0.598
Verrucomicrobiota	0.01	0.23	0.108	0.325

1) CON indicates the control group, which was fed a basal diet.

2) HDMF represents the treatment group, which was received an additional 1.0 g per day of 4-hydroxy-2,5-dimethyl-3(2H)-furanone (HDMF) on top of the basal diet.

SEM, standard error of the mean.

**Table 7:** Effect of dietary HDMF supplementation on the rumen bacterial composition (genus, >0.50%) of Hu sheep

Genus name	CON^1)^	HDMF^2)^	SEM	p-value
*Prevotella*	32.04	33.46	2.906	0.734
*Succinivibrio*	15.72	11.18	3.522	0.398
*Succinivibrionaceae UCG-001*	6.30	4.57	2.050	0.561
*Clostridia UCG-014*	4.80	5.26	1.345	0.811
*Roseburia*	3.08	4.62	1.329	0.441
*Oribacterium*	3.90	2.66	0.978	0.388
*Selenomonas*	0.97	4.52	2.040	0.324
*Dialister*	1.46	3.28	0.844	0.188
*Succiniclasticum*	2.25	2.33	0.682	0.933
*Ruminococcus*	1.75	1.76	0.659	0.995
*Rikenellaceae RC9 gut group*	0.96	2.35	0.678	0.175
*Muribaculaceae*	0.99	1.69	0.497	0.381
*Lachnospiraceae NK3A20 group*	1.68	0.96	0.324	0.159
*Prevotellaceae UCG-001*	0.66	1.90	0.432	0.110
*Eubacterium coprostanoligenes group*	0.82	1.30	0.443	0.511
*Erysipelotrichaceae UCG-002*	2.04	0.08	0.630	0.144
*Lachnospira*	1.26	0.84	0.269	0.294
*Olsenella*	0.77	0.76	0.242	0.957
*Eubacterium eligens group*	1.08^a^	0.37^b^	0.187	0.017
*Syntrophococcus*	0.89	0.47	0.182	0.152
*Candidatus Amoebophilus*	1.25	0.02	0.621	0.341
*RF39*	0.78	0.43	0.130	0.093
*Erysipelotrichaceae UCG-009*	0.39	0.79	0.320	0.418
*Ruminococcus gauvreauii group*	0.60	0.41	0.201	0.536

1) CON indicates the control group, which was fed a basal diet.

2) HDMF represents the treatment group, which was received an additional 1.0 g per day of 4-hydroxy-2,5-dimethyl-3(2H)-furanone (HDMF) on top of the basal diet.

a,b In the same row, values with different superscripts differ significantly (p<0.05).

SEM, standard error of the mean.

**Table 8:** Effect of dietary HDMF supplementation on predicted metabolic pathways of ruminal bacterial microbiome of Hu sheep

Item	CON^1)^	HDMF^2)^	SEM	p-value
Metabolism of cofactors and vitamins	14.10	14.46	0.167	0.151
Carbohydrate metabolism	14.09	14.05	0.140	0.856
Amino acid metabolism	13.12	13.19	0.071	0.485
Metabolism of terpenoids and polyketides	9.20	8.95	0.137	0.211
Replication and repair	6.68	6.68	0.062	0.984
Metabolism of other amino acids	6.57	6.60	0.077	0.774
Energy metabolism	5.84	5.70	0.069	0.173
Glycan biosynthesis and metabolism	5.02	5.33	0.126	0.117
Lipid metabolism	4.25	4.09	0.084	0.200
Translation	3.70	3.69	0.030	0.949
Folding, sorting and degradation	3.25	3.23	0.056	0.870
Cell motility	3.13	2.77	0.189	0.197
Nucleotide metabolism	2.22	2.24	0.020	0.493
Biosynthesis of other secondary metabolites	2.17	2.26	0.115	0.573
Cell growth and death	1.74	1.76	0.013	0.199
Membrane transport	1.67	1.60	0.048	0.325
Transcription	1.18	1.22	0.033	0.369
Xenobiotics biodegradation and metabolism	0.38	0.49	0.168	0.637
Cellular community - prokaryotes	0.35	0.32	0.030	0.418
Signal transduction	0.33	0.31	0.012	0.323
Environmental adaptation	0.28	0.26	0.010	0.202
Transport and catabolism	0.25	0.26	0.007	0.128
Infectious disease: bacterial	0.18	0.18	0.003	0.666

1) CON indicates the control group, which was fed a basal diet.

2) HDMF represents the treatment group, which was received an additional 1.0 g per day of 4-hydroxy-2,5-dimethyl-3(2H)-furanone (HDMF) on top of the basal diet.

SEM, standard error of the mean.
